# Trends in Stroke Incidence in High-Income Countries in the 21st Century: Population-Based Study and Systematic Review

**DOI:** 10.1161/STROKEAHA.119.028484

**Published:** 2020-03-25

**Authors:** Linxin Li, Catherine A. Scott, Peter M. Rothwell

**Affiliations:** From the Nuffield Wolfson Centre for Prevention of Stroke and Dementia, Nuffield Department of Clinical Neurosciences, University of Oxford, United Kingdom.

**Keywords:** cohort study, incidence, stroke, systematic review

## Abstract

Population-based studies provide the most reliable data on stroke incidence. A previous systematic review of population-based studies suggested that stroke incidence in high-income countries decreased by 42% between the 1970s and early 2000s. However, it is uncertain whether this trend of steady decline has been maintained in more recent periods.

**Methods—:**

Data from OCSP (Oxfordshire Community Stroke Project; 1981–1986) and OXVASC (Oxford Vascular Study; 2002–2017) along with other published population-based stroke incidence studies that reported temporal trends of stroke incidence since 1990 in high-income countries were included. Age-standardized relative incidence rate ratios were calculated for each study and then pooled with inverse-variance weighted random-effects meta-analysis. Projection estimates were calculated for the number of incident stroke patients in the United Kingdom from year 2015 to 2045.

**Results—:**

In Oxfordshire, stroke incidence fell by 32% from OCSP to OXVASC, with a similar trend before or after year 2000. With the projected aging population, if the age-specific stroke incidence continued to decrease at its current rate (6% every 5 years), there would still be a 13% increase of the number of first-ever strokes in the United Kingdom up to year 2045. Incorporating the Oxfordshire data with other 12 population-based studies, stroke incidence declined steadily between the 1990s and 2010s within each study, resulting in a 28% decline over an average period of 16.5 years (pooled incidence rate ratio, 0.72 [95% CI, 0.66–0.79]; *P*<0.0001). The trend was the same for men (0.69 [95% CI, 0.61–0.77]; *P*<0.0001) and women (0.66 [95% CI, 0.59–0.74]; *P*<0.0001) and remained consistent after year 2010 in OXVASC. Proportion of disabling or fatal stroke also decreased over time (early versus later period, 53.6% versus 46.1%; *P*=0.02).

**Conclusions—:**

Stroke incidence is continuing to decline with steady rate in Oxfordshire and in other high-income settings. However, the absolute number of strokes occurring is not falling.

## Abstract 2

Supplemental Digital Content is available in the text.

Stroke is the second leading cause of death worldwide and the main cause of long-term neurological disability in adults.^1^ In high-income countries, increased use of preventive treatments and major reductions in premorbid risk factors between the 1970s and early 2000s have coincided with significant falls in stroke incidence during this period.^2^ However, it is uncertain whether this trend of steady decline has been maintained in more recent years, particularly in view of the continuing aging of the population and the rise of diabetes mellitus and obesity in recent decades.^3–5^ Reliable estimates and projections of the future stroke burden are important for long-term healthcare infrastructure planning, policy making, and are important for the organization of stroke services and prevention activities.

Some studies based on administrative data reported that the reduction of stroke incidence was less marked in the early 2000s.^6,7^ However, accuracy of coding of stroke in routinely collected data can also vary over time.^8,9^ Prospective population-based incidence studies provide the most reliable data on stroke incidence.^2^ Therefore, in a population-based study in Oxfordshire, United Kingdom, we determined the change of stroke incidence in the last 4 decades and estimated the potential burden of stroke incidence in the United Kingdom from year 2015 to 2045. A few other population-based studies also maintained ideal case-ascertainment procedures over a long period of time to provide comparable estimates of stroke incidence change over time. Since the publication of the most recent systematic review of temporal trend of stroke incidence in high-income countries,^10^ several population-based studies have reported new data on stroke incidence, particularly after year 2010.^11–18^ We, therefore, also combined our results in an updated systematic review to determine the temporal trends of stroke incidence between 1990s and 2010s in high-income settings.

## Methods

Requests for access to data from the OXVASC (Oxford Vascular Study) will be considered by the corresponding author.

### Study Population

OXVASC is an ongoing prospective population-based study of the incidence and outcome of all acute vascular events in a mixed rural/urban population. The study population comprises all 92 728 individuals, irrespective of age, registered with ≈100 general practitioners in 9 general practices in Oxfordshire, United Kingdom. Ascertainment of acute vascular events started on April 1, 2002, and is ongoing. This analysis reports all first-ever incident strokes during the first 15 years (2002–2017).

The detailed study methodology and case definitions have been published before (Methods in the Data Supplement), and multiple overlapping methods of hot and cold pursuit were used to achieve near complete and consistent ascertainment of all individuals with stroke.^19,20^ To ensure consistency, one senior stroke neurologist (P.M.R.) has prospectively reviewed all cases over the 15 years. Stroke was defined as an event with appropriate symptoms lasting >24 hours.

Stroke incidence in OXVASC was also compared with that in OCSP (Oxfordshire Community Stroke Project), which was a high-quality, population-based study of all first-ever strokes in 1981 to 1986 in the same general practice population. The methodology of OCSP has also been published before (Methods in the Data Supplement),^21^ and the case diagnosis, assessment, and follow-up were similar to those in OXVASC. In particular, to ensure consistency of clinical diagnosis between OCSP and OXVASC, summaries of all potential cases in the first 2 years of OXVASC were reviewed by the principal investigator of OCSP to ensure that the application of definitions of strokes was comparable between the 2 studies.^19^

In both OCSP and OXVASC, patients were ascertained and assessed face-to-face by study physicians as soon as possible after the initial presentation in hospital, an emergency clinic, or at home. If a patient died before assessment, we obtained an eyewitness account of the clinical event and reviewed any relevant clinical records. In OCSP, brain computed tomography or autopsy was the first-line imaging modality, and 12% of the cases did not have brain imaging or autopsy. In OXVASC, patients routinely had brain imaging (computed tomography or magnetic resonance), vascular imaging (carotid Doppler or computed tomography angiography/magnetic resonance angiography or digital subtraction angiography), 12-lead electrocardiography, and standard blood tests. Echocardiography, 24-hour ECG (Holter), and 5-day ambulatory ECG monitoring were done when clinically indicated. Less than 5% of the cases in OXVASC did not have any brain imaging or autopsy.

All patients were followed up face-to-face at 1, 6, 12, 60, and 120 months by a study nurse or physician to determine their functional status (modified Rankin Scale [mRS]).^22^ Disabling or fatal stroke is defined as a new disability (mRS score, >2) or death or progression of disability (1 score increase in mRS) in those with premorbid disability (premorbid mRS score, >2) using the 1-month mRS. For patients who had moved out of the study area, follow-up was performed by telephone or email. All patients were flagged for the Office for National Statistics mortality data, and all deaths during follow-up were recorded with causes.

### Systematic Review

We followed the PRISMA guideline and identified all stroke incidence and prevalence studies referenced in previous published reviews and hand-searched PUBMED and EMBASE for any follow-up or secondary studies using the author and study names from the primary studies. We also performed a further search of PUBMED (1950 to December 2017) and EMBASE (1974 to December 2017) using the following search terms without restrictions: “exp STROKE/” or “first ever stroke*.ti,ab.” and “incidence/” or “registries/.” The abstracts of all articles identified from initial searches were reviewed by 2 authors (L.L. and C.A.S.), and both authors reviewed the full text of all eligible studies. In cases of disagreement about the eligibility of studies or data extraction, consensus was reached through joint reassessment.

Studies were included in the main analysis if they fulfilled the following criteria: (1) published incidence studies satisfying the quality criteria of ideal population-based study^23,24^; (2) conducted in high-income countries^10^; (3) reported stroke incidence, incidence rate ratio (IRR) or raw numbers sufficient to calculate incidence, or change in incidence at ≥2 time periods; (4) reported stroke incidence between 1990 (±5 years) and 2017, with the latest time period after 2010; and (5) English language. Studies that fulfilled criterias 1 to 3 and reported the latest time point between year 2000 and 2010 were also included in subgroup analyses. Population-based studies confined to only one pathological stroke subtype were excluded. Where there was >1 publication on a cohort of patients, data on incidence rate (IR) were taken from the most recent publication or the publication with the most complete raw numbers. Updated data from OXVASC were also included.

Information was extracted from each report on the population studied, study period (duration), observed person-years, and the total number of incident stroke cases. Where possible, we also documented the reported numbers of incident stroke cases by sex, stroke subtype (ischemic stroke, intracerebral hemorrhage, and subarachnoid hemorrhage), and stroke severity (disabling/fatal versus nondisabling). Where available, crude and standardized IRs with CIs, as well as raw numbers (numerator, n, and denominator, N), were extracted for each time period reported. Where IR, n, or N were not reported, when possible, these were calculated based on reported IRR.

### Statistical Analyses

For Oxfordshire and other included studies, age-specific crude IRs (per 100 000 population/year) were calculated with CIs estimated assuming a Poisson distribution. For studies that reported stroke incidence in different age groups, sexes, ethnicities, or stroke subgroups, where raw numbers were not available, we used inverse-variance weighted fixed-effects meta-analysis to generate summary IRs.

All crude IRs were standardized to the European population using the direct method.^25^ Standardized relative IRRs were then calculated for the chosen 2 periods within each study using Poisson regression models adjusting for the age structure of the 2 populations. IRRs from individual studies were then pooled with inverse-variance weighted random-effects meta-analysis to generate a pooled IRR with 95% CIs. We estimated the percentage of variability across studies attributable to heterogeneity beyond chance using the I^2^ statistic.

In the main analysis, pooled estimates for temporal trends of stroke incidence between 1990s and 2010s were calculated. We also performed subgroup analyses stratified by stroke subtypes (ischemic stroke, intracerebral hemorrhage, and subarachnoid hemorrhage), sex, stroke severity (disabling/fatal versus nondisabling), and study periods (1990–2000 versus after year 2000). Sensitivity analyses were also performed in relation to study duration.

Finally, to estimate the number of incident stroke patients on a national basis in the United Kingdom from 2015 up to 2045, we used the age-specific incidence during 2014 to 2017 in OXVASC for standardization to the projected UK population.^26^ Two different scenarios for changes in age-specific incidence were used: stable rates over time or maintaining the current 6% decrease every 5 years. Moreover, we calculated how much the incidence would have to decrease every 5 years to maintain a stable number of incident stroke patients in the United Kingdom in 2045 compared with 2015.

All analyses were performed using SPSS, version 22.

### Standard Protocol Approvals, Registrations, and Patient Consents

Written informed consent or assent from relatives was obtained in all participants. OXVASC was approved by the local research ethics committee (OREC A: 05/Q1604/70).

## Results

Based on 2811 incident stroke cases in OCSP (n=557) and OXVASC (n=2254), stroke incidence fell by 32% from 1981 to 1986 to 2014 to 2017 (IRR, 0.68 [95% CI, 0.60–0.78]; *P*<0.0001), with no evidence of heterogeneity before and after the year 2000 (*P*_het_=0.54; OXVASC 2002–2005 versus OCSP 1981–1986: 0.80 [95% CI, 0.70–0.91]; *P*=0.0006), and after (2014–2017 versus 2002–2005: 0.85 [95% CI, 0.74–0.98]; *P*=0.03).

Using the above estimates, we applied different scenarios for the projection of the number of first-ever stroke patients in the United Kingdom between 2015 and 2045. The UK population is projected to increase by 3.6 million (5.5%) over the next 10 years and reaching 72.9 million in mid-2041 with doubling number of individuals aged ≥85 years.^26^ If the age-specific incidence remained stable over the next 30 years, the number of incident stroke would increase by 66% from year 2015 to 2045. If the age-specific stroke incidence continued to decline with its current magnitude (OXVASC estimates: 6% every 5 years), there would be a 13% increase of the number of first-ever strokes in the United Kingdom up to year 2045. To maintain a stable number of incident stroke patients in the United Kingdom in 2050, the incidence would have to decrease by 8% every 5 years.

Sixteen thousand six hundred thirty-eight citations were identified, and 13 population-based studies from 9 high-income countries were included in the systematic review (Figure I and References in the Data Supplement).^11–16,19,27–29,30–31^ Of the 13 studies, 9 (including unpublished data from OXVASC)^11–19,29–31^ reported temporal trends of stroke incidence including at least 1 period after year 2010 and were thus included in the main analysis (Table 1). An additional 4 studies reporting either temporal trends between 1990 and 2000 or after year 2000 were also included in the sensitivity analysis (Table I in the Data Supplement). No published study reported change of stroke incidence beyond year 2010.

**Table 1.**
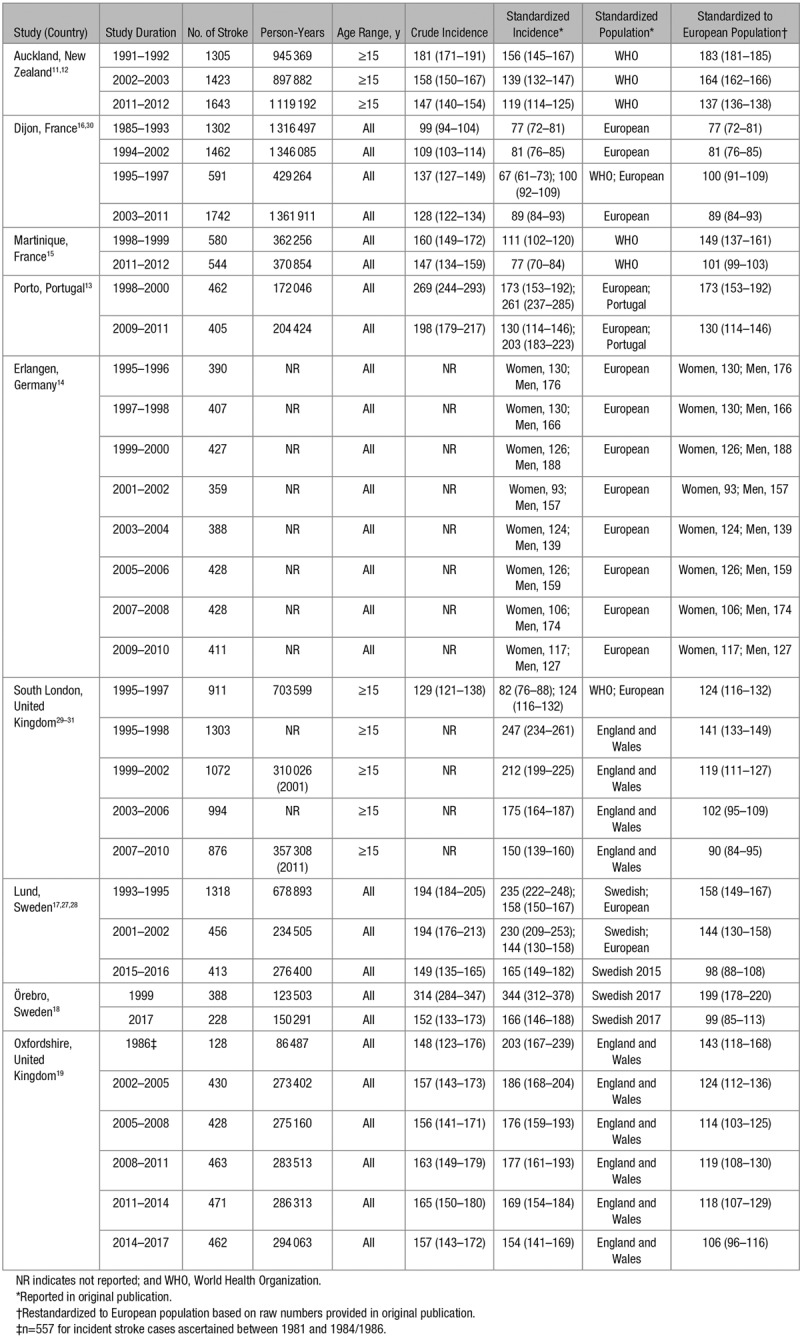
Study Characteristics and Incidence (95% CI) of First-Ever Stroke (per 100 000 Population) in Population-Based Studies Included in Main Analyses Stratified by Study Periods

Combining results from Oxfordshire and the systematic review, 31 351 new stroke cases occurred during ≈18 453 235 person-years of observation. Despite noticeable variation in absolute age-standardized stroke IRs between studies, stroke incidence declined significantly between the 1990s and 2010s in all studies (Table 1; Table I in the Data Supplement), resulting in less between-study difference in absolute rates after year 2010 (highest versus lowest incidence before year 2000: 183 versus 81 per 100 000 population; after year 2010: 143 versus 98 per 100 000 population).

**Figure 1.**
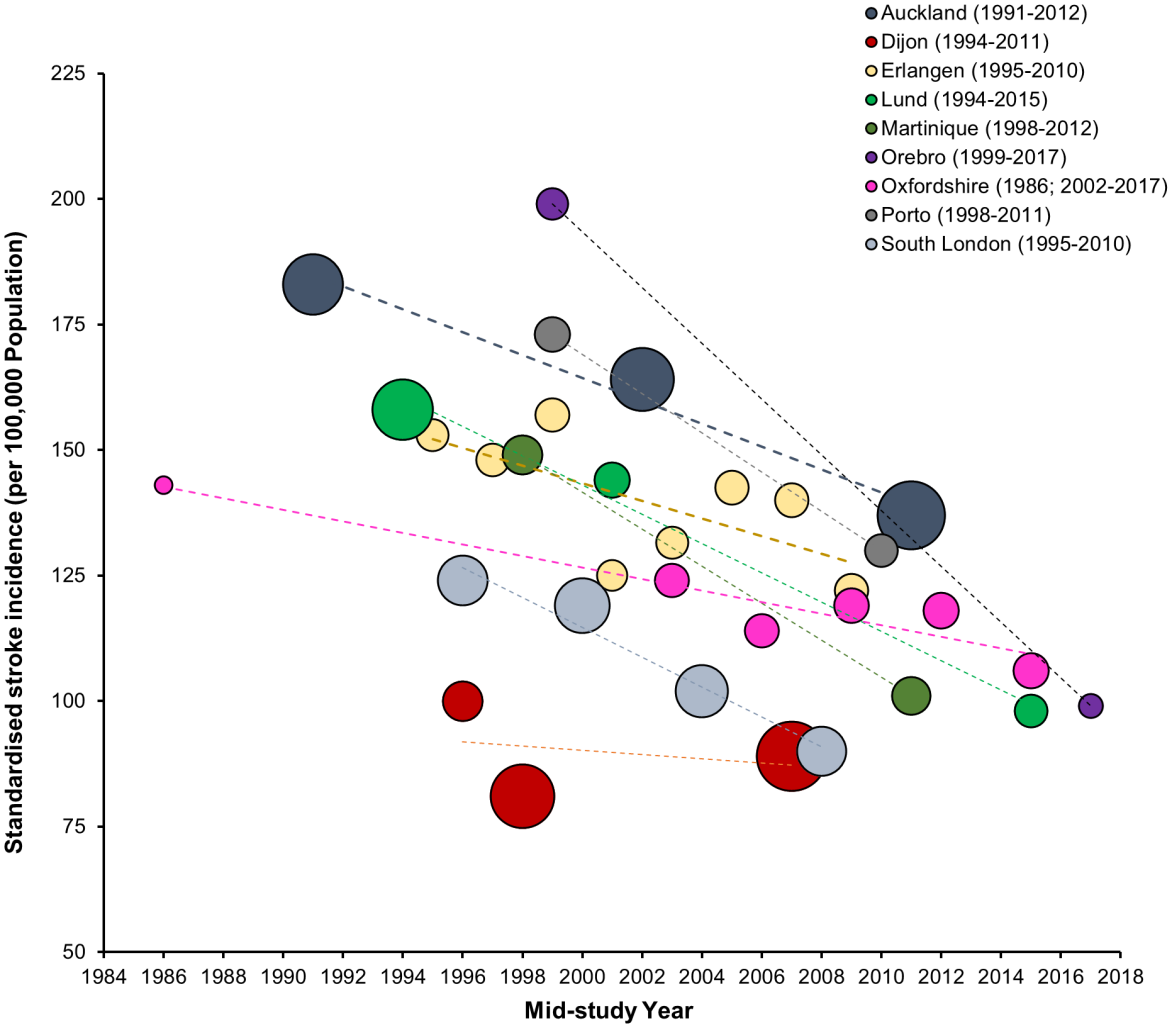
Temporal trends of standardized stroke incidence in population-based studies reporting at least 1 time point after year 2010 (1990–2010).

**Figure 2.**
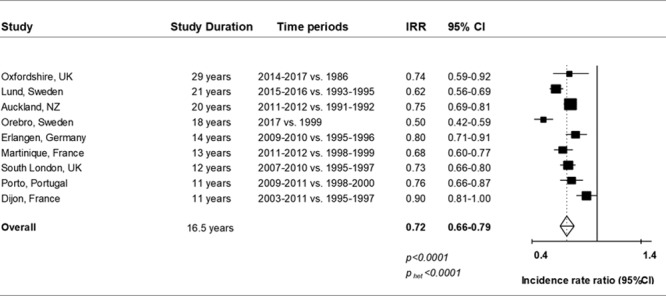
Meta-analysis (random effects) of standardized incidence rate ratio (IRR; temporal trend) in population-based studies of first-ever stroke (1990–2010). †Raw numbers of incident stroke cases for each study for the 2 time periods included are presented in Table 1.

Although the rates of reduction in stroke incidence varied slightly between studies, the trend of steady decline was consistent across studies (Figure 1). Consequently, the pooled estimate from the 9 studies between 1990s and 2010s suggested a 28% decline of stroke incidence during an average study duration of 16.5 years (IRR, 0.72 [95% CI, 0.66–0.79]; *P*<0.0001; Figure 2), with no differences between men and women (8 studies; *P*_difference_=0.56; Table 2). The results were also largely consistent by stroke subtypes (Table 2). Sensitivity analyses excluding the 4 studies with the longest study duration (Oxfordshire, Sweden,^17,18,32,33^ and Auckland^11,12^) also showed consistent results (mean study duration, 12 years; IRR, 0.78 [95% CI, 0.70–0.86]; *P*<0.0001).

**Table 2.**
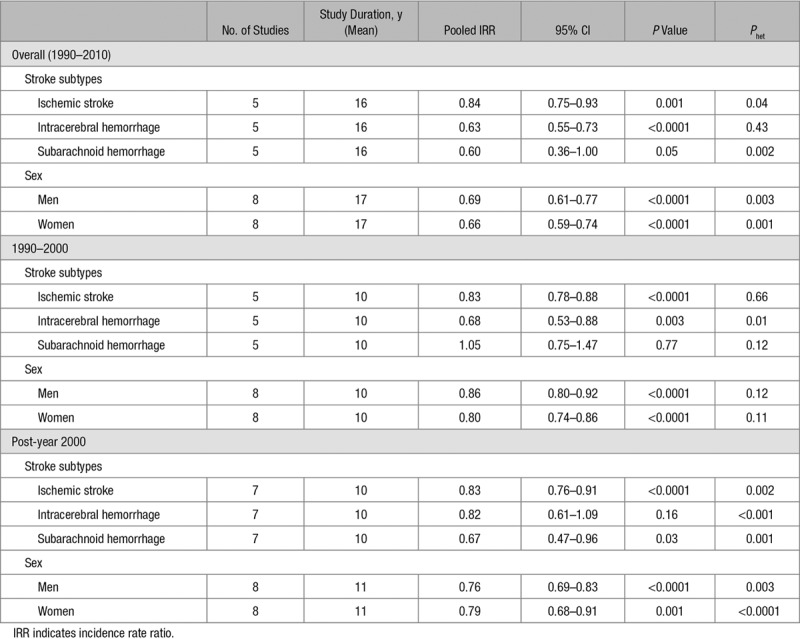
Meta-Analysis (Random Effects) of Standardized IRR (Temporal Trend) in Population-Based Studies of First-Ever Stroke Stratified by Stroke Subtypes and by Sex

**Figure 3.**
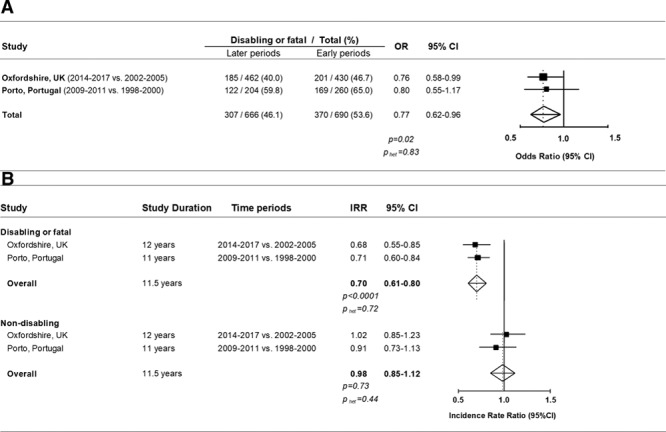
Meta-analysis (random effects) of standardized incidence rate ratio (IRR; temporal trend) in population-based studies of first-ever stroke stratified by stroke severity. **A**, Change of the proportions of disabling or fatal stroke. **B**, Change of IRR. OR indicates odds ratio. †Raw numbers of incident stroke cases for each study for the 2 time periods included can be derived from **A**.

Two studies of similar duration (Oxfordshire and Porto^13^) reported the temporal trends of stroke incidence stratified by stroke severity (Figure 3). In contrast to a 30% reduction of disabling or fatal stroke during a mean study duration of 11.5 years, there was no statistically significant reduction in nondisabling stroke (pooled IRR, 0.98 [95% CI, 0.85–1.12]; *P*=0.73; Figure 3) due apparently to reductions in the proportion of disabling or fatal stroke (2 studies; early versus later period, 53.6% versus 46.1%; odds ratio, 0.77 [95% CI, 0.62–0.96]; *P*=0.02; Figure 3).

In the subgroup analysis stratified by study periods, although more heterogeneity was observed post-year 2000 (Figure II in the Data Supplement), the pooled estimates for the reduction of stroke incidence in each period were consistent (1990–2000: 9 studies; mean study duration, 9 years; IRR, 0.84 [95% CI, 0.74–0.94]; *P*=0.003 versus post-year 2000: 11 studies; mean study duration, 10 years; IRR, 0.82 [95% CI, 0.73–0.93]; *P*=0.001; Figure II in the Data Supplement) and were maintained after year 2010 in OXVASC (Figure 1). There was also no difference in the trends between men and women within each period (*P*_difference_=0.22 for 1990–2000 and *P*_difference_=0.73 post-year 2000; Table 2). Again, results were also largely comparable for ischemic versus hamorrhagic strokes, although the reduction of subarachnoid hemorrhage was more marked post-year 2000 (Table 2), whereas the reduction of intracerebral hemorrhage became less marked in more recent periods (Table 2).

## Discussion

In this updated systematic review of all population-based stroke incidence studies in high-income countries, we showed that the previously suggested 1.1% yearly percentage reduction in stroke incidence from the 1970s to early 2000s^2^ is maintained and stroke incidence continued to decline at an annual rate of 1.0% to 1.5% in the last 3 decades. However, with the aging population, even if the age-specific stroke incidence continued to decrease at its current rate, there would still be a 13% increase of the number of first-ever strokes in the United Kingdom in year 2045.

Our findings based on population-based stroke incidence studies in high-income countries are supported by data from hospital-based registries, which also show a steady decline of hospitalized stroke in recent years in southeast Asia,^34^ Western Europe,^35–37^ and North America.^38–40^ The results are also consistent with the data modeling results from the Global Burden of Disease analysis.^5^ Moreover, it is encouraging that the previously suggested 1.1% yearly percentage reduction in stroke incidence from the 1970s to early 2000s was maintained,^2^ possibly due to continued effort in implementing preventive treatment at the population level to reduce smoking, hypertension, and other vascular risk factors.^19,36,41,42^

However, even if stroke incidence continued to decline at its current rate, there would still be a 13% increase of the number of new strokes in the United Kingdom in year 2045 due to the aging population. Similar projections have also been reported for other European countries.^27,32,33^ Moreover, reduced stroke case fatality in high-income countries would lead to growing numbers of stroke survivors, contributing to the increase of the overall burden of stroke.^10^

There was variation in the annual reduction rates between studies, with Dijon^16^ showing the least steep slope and Örebro,^18^ Martinique,^15^ and Porto^13^ showing the most prominent reduction. In other studies based on administrative data, one Swedish study and 2 Japanese registries also reported less marked reduction rates in the early 2000s than those from other high-income countries.^6,7,43^ The overall difference in the time trends may reflect the difference by stroke subtypes between studies. While some studies found consistent decline for all types of strokes,^13,15^ others reported diverging trend for ischemic stroke versus hemorrhagic stroke.^12,17,19,44–46^ Moreover, although consistent decline for men and women was found in our pooled analysis and also reported in other hospital-based registries,^39^ the Erlangen study only found a significant decline for men.^14^ Given that the included population-based studies had broadly similar ascertainment methods and also maintained consistent methodology over time, the observed differences between studies would appear to be real and may be accounted for by differences in life expectancy, risk factor prevalence, control of risk factors, and accessibility of health services.^30^

Notably, in contrast to a steady decline of stroke incidence for ischemic stroke, our estimate suggested less marked change of stroke incidence for intracerebral hemorrhage in more recent years. This apparent divergent trend could perhaps be explained by increasing use of antithrombotic treatment at older ages, especially with the increasing burden of atrial fibrillation.^44^

Both OXVASC and the Porto stroke registry^13^ found no significant reduction in nondisabling stroke in the past 10 years. Cerebrovascular events could have become less severe due to implementation of preventive treatment or less disabling due to improved acute stroke care. On the other hand, increasing public awareness of stroke symptoms and growing use of neuroimaging and diagnostic awareness among healthcare providers might have also resulted in improving ascertainment of minor strokes over time.

Although we consider our findings to be valid, our study has limitations. First, although we confined our analysis to high-quality population-based stroke incidence studies that maintained the ideal methodology over time, completeness of ascertainment might have changed over time.^47^ However, we found a consistent and steady decline across all studies. Second, we only had aggregated data from published results and were, therefore, unable to assess thoroughly potential reasons for the observed overall temporal trends or the heterogeneity between studies, particularly if any demographic change, such as change in occupation or standard of living, had any role in explaining the observed trends across studies. Third, proportion of unclassified stroke differed considerably between studies, with further variation over time. Therefore, it was difficult to provide accurate pooled estimates comparing the temporal change of incidence in ischemic versus hemorrhagic strokes. Moreover, most studies did not provide data on the change of etiological subtypes of ischemic strokes over time. We were also unable to compare the incidence change by stroke etiology. However, one recent systematic review of population- and hospital-based studies suggested an increasing trend of cardioembolic stroke and a decrease for lacunar stroke in high-income countries in the last 30 years.^48^

In conclusion, stroke incidence is continuing to decline in Oxfordshire and in other high-income settings in the last 30 years. However, with the aging population, even if the age-specific stroke incidence continued to decrease at its current rate, the number of new stroke cases annually in high-income countries would continue to increase in the next 30 years.

## Acknowledgments

We are grateful to all the staff in the general practices who collaborated in the Oxford Vascular Study.

## Sources of Funding

The Oxford Vascular Study is funded by the National Institute for Health Research Oxford Biomedical Research Centre, Wellcome Trust, Wolfson Foundation, and British Heart Foundation. L. Li receives a fellowship award from the Medical Research Foundation.

## Disclosures

None.

## Supplementary Material



## References

[1] Strong K, Mathers C, Bonita R (2007). Preventing stroke: saving lives around the world.. Lancet Neurol.

[2] Feigin VL, Lawes CM, Bennett DA, Barker-Collo SL, Parag V (2009). Worldwide stroke incidence and early case fatality reported in 56 population-based studies: a systematic review.. Lancet Neurol.

[3] Di Cesare M, Bentham J, Stevens GA, Zhou B, Danaei G, Lu Y (2016). Trends in adult body-mass index in 200 countries from 1975 to 2014: a pooled analysis of 1698 population-based measurement studies with 19.2 million participants.. Lancet.

[4] Danaei G, Finucane MM, Lu Y, Singh GM, Cowan MJ, Paciorek CJ, Global Burden of Metabolic Risk Factors of Chronic Diseases Collaborating Group (Blood Glucose) (2011). National, regional, and global trends in fasting plasma glucose and diabetes prevalence since 1980: systematic analysis of health examination surveys and epidemiological studies with 370 country-years and 2·7 million participants.. Lancet.

[5] Johnson CO, Nguyen M, Roth GA, Nichols E, Alam T, Abate D (2019). Global, regional, and national burden of stroke, 1990-2016: a systematic analysis for the global burden of disease study 2016.. Lancet Neurol.

[6] Harmsen P, Wilhelmsen L, Jacobsson A (2009). Stroke incidence and mortality rates 1987 to 2006 related to secular trends of cardiovascular risk factors in Gothenburg, Sweden.. Stroke.

[7] Hata J, Ninomiya T, Hirakawa Y, Nagata M, Mukai N, Gotoh S (2013). Secular trends in cardiovascular disease and its risk factors in Japanese: half-century data from the Hisayama Study (1961-2009).. Circulation.

[8] Li L, Binney LE, Luengo-Fernandez R, Silver LE, Rothwell PM (2019). Temporal trends in the accuracy of hospital diagnostic coding for identifying acute stroke: a population-based study.. Eur Stroke J.

[9] Aboa-Eboulé C, Mengue D, Benzenine E, Hommel M, Giroud M, Béjot Y (2013). How accurate is the reporting of stroke in hospital discharge data? A pilot validation study using a population-based stroke registry as control.. J Neurol.

[10] Feigin VL, Forouzanfar MH, Krishnamurthi R, Mensah GA, Connor M, Bennett DA, Global Burden of Diseases, Injuries, and Risk Factors Study 2010 (GBD 2010) and the GBD Stroke Experts Group (2014). Global and regional burden of stroke during 1990-2010: findings from the Global Burden of Disease Study 2010.. Lancet.

[11] Feigin VL, Krishnamurthi RV, Barker-Collo S, McPherson KM, Barber PA, Parag V, ARCOS IV Group (2015). 30-year trends in stroke rates and outcome in Auckland, New Zealand (1981-2012): a Multi-Ethnic Population-Based Series of Studies.. PLoS One.

[12] Krishnamurthi RV, Barker-Collo S, Parag V, Parmar P, Witt E, Jones A (2018). Stroke incidence by major pathological type and ischemic subtypes in the Auckland Regional Community Stroke Studies: changes between 2002 and 2011.. Stroke.

[13] Correia M, Magalhães R, Felgueiras R, Quintas C, Guimarães L, Silva MC (2017). Changes in stroke incidence, outcome, and associated factors in Porto between 1998 and 2011.. Int J Stroke.

[14] Kolominsky-Rabas PL, Wiedmann S, Weingärtner M, Liman TG, Endres M, Schwab S (2015). Time trends in incidence of pathological and etiological stroke subtypes during 16 years: the erlangen stroke project.. Neuroepidemiology.

[15] Olindo S, Chausson N, Mejdoubi M, Jeannin S, Rosillette K, Saint-Vil M (2014). Trends in incidence and early outcomes in a black afro-caribbean population from 1999 to 2012: etude réalisée en martinique et centrée sur l’Incidence des Accidents Vasculaires Cérébraux II Study.. Stroke.

[16] Béjot Y, Daubail B, Jacquin A, Durier J, Osseby GV, Rouaud O (2014). Trends in the incidence of ischaemic stroke in young adults between 1985 and 2011: the Dijon Stroke Registry.. J Neurol Neurosurg Psychiatry.

[17] Aked J, Delavaran H, Norrving B, Lindgren A (2018). Temporal trends of stroke epidemiology in Southern Sweden: a Population-Based Study on stroke incidence and early case-fatality.. Neuroepidemiology.

[18] Appelros P (2019). Secular trends of stroke epidemiology in orebro, sweden, 2017 compared to the trends in 1999: a population-based study.. Cerebrovasc Dis.

[19] Rothwell PM, Coull AJ, Giles MF, Howard SC, Silver LE, Bull LM, Oxford Vascular Study (2004). Change in stroke incidence, mortality, case-fatality, severity, and risk factors in Oxfordshire, UK from 1981 to 2004 (Oxford Vascular Study).. Lancet.

[20] Rothwell PM, Coull AJ, Silver LE, Fairhead JF, Giles MF, Lovelock CE, Oxford Vascular Study (2005). Population-based study of event-rate, incidence, case fatality, and mortality for all acute vascular events in all arterial territories (Oxford Vascular Study).. Lancet.

[21] Bamford J, Sandercock P, Dennis M, Warlow C, Jones L, McPherson K (1988). A prospective study of acute cerebrovascular disease in the community: the Oxfordshire Community Stroke Project 1981-86. 1. Methodology, demography and incident cases of first-ever stroke.. J Neurol Neurosurg Psychiatry.

[22] van Swieten JC, Koudstaal PJ, Visser MC, Schouten HJ, van Gijn J (1988). Interobserver agreement for the assessment of handicap in stroke patients.. Stroke.

[23] Feigin VL, Lawes CM, Bennett DA, Anderson CS (2003). Stroke epidemiology: a review of population-based studies of incidence, prevalence, and case-fatality in the late 20^th^ century.. Lancet Neurol.

[24] Sudlow CL, Warlow CP (1997). Comparable studies of the incidence of stroke and its pathological types: results from an international collaboration. International stroke incidence collaboration.. Stroke.

[25] Doll R, Cook P (1967). Summarizing indices for comparison of cancer incidence data.. Int J Cancer.

[26] Statistics OfN National population projections: 2016-based statistical bulletin.. https://www.ons.gov.uk/releases/nationalpopulationprojections2016basedstatisticalbulletin.

[27] Hallström B, Jönsson AC, Nerbrand C, Norrving B, Lindgren A (2008). Stroke incidence and survival in the beginning of the 21^st^ century in southern Sweden: comparisons with the late 20^th^ century and projections into the future.. Stroke.

[28] Johansson B, Norrving B, Lindgren A (2000). Increased stroke incidence in Lund-Orup, Sweden, between 1983 to 1985 and 1993 to 1995.. Stroke.

[29] Wolfe CD, Rudd AG, Howard R, Coshall C, Stewart J, Lawrence E (2002). Incidence and case fatality rates of stroke subtypes in a multiethnic population: the South London Stroke Register.. J Neurol Neurosurg Psychiatry.

[30] Wolfe CD, Giroud M, Kolominsky-Rabas P, Dundas R, Lemesle M, Heuschmann P (2000). Variations in stroke incidence and survival in 3 areas of Europe. European Registries of Stroke (EROS) collaboration.. Stroke.

[31] Heuschmann PU, Grieve AP, Toschke AM, Rudd AG, Wolfe CD (2008). Ethnic group disparities in 10-year trends in stroke incidence and vascular risk factors: the South London Stroke Register (SLSR).. Stroke.

[32] Truelsen T, Piechowski-Jóźwiak B, Bonita R, Mathers C, Bogousslavsky J, Boysen G (2006). Stroke incidence and prevalence in Europe: a review of available data.. Eur J Neurol.

[33] Béjot Y, Bailly H, Durier J, Giroud M (2016). Epidemiology of stroke in Europe and trends for the 21^st^ century.. Presse Med.

[34] Tan CS, Müller-Riemenschneider F, Ng SH, Tan PZ, Chan BP, Tang KF, Singapore Stroke Registry (2015). Trends in stroke incidence and 28-day case fatality in a nationwide stroke registry of a multiethnic asian population.. Stroke.

[35] Iacoviello L, Costanzo S, Persichillo M, Sparano A, Bartolo M, Polizzi BM (2016). Hospital-based register of stroke in the molise region: focus on main subtypes of stroke. Years 2009-2013.. Neurol Sci.

[36] Santalucia P, Baviera M, Cortesi L, Tettamanti M, Marzona I, Nobili A (2015). Epidemiologic trends in hospitalized ischemic stroke from 2002 to 2010: results from a Large Italian Population-Based Study.. J Stroke Cerebrovasc Dis.

[37] Lewsey JD, Jhund PS, Gillies M, Chalmers JW, Redpath A, Kelso L (2009). Age- and sex-specific trends in fatal incidence and hospitalized incidence of stroke in Scotland, 1986 to 2005.. Circ Cardiovasc Qual Outcomes.

[38] Khan NA, McAlister FA, Pilote L, Palepu A, Quan H, Hill MD (2017). Temporal trends in stroke incidence in South Asian, Chinese and white patients: a population based analysis.. PLoS One.

[39] Koton S, Schneider AL, Rosamond WD, Shahar E, Sang Y, Gottesman RF (2014). Stroke incidence and mortality trends in US communities, 1987 to 2011.. JAMA.

[40] Carandang R, Seshadri S, Beiser A, Kelly-Hayes M, Kase CS, Kannel WB (2006). Trends in incidence, lifetime risk, severity, and 30-day mortality of stroke over the past 50 years.. JAMA.

[41] Vangen-Lønne AM, Wilsgaard T, Johnsen SH, Løchen ML, Njølstad I, Mathiesen EB (2017). Declining incidence of ischemic stroke: what is the impact of changing risk factors? The Tromsø Study 1995 to 2012.. Stroke.

[42] Collaboration NCDRF (2017). Worldwide trends in blood pressure from 1975 to 2015: a pooled analysis of 1479 population-based measurement studies with 19.1 million participants.. Lancet.

[43] Kita Y, Turin TC, Ichikawa M, Sugihara H, Morita Y, Tomioka N (2009). Trend of stroke incidence in a Japanese population: takashima stroke registry, 1990-2001.. Int J Stroke.

[44] Lovelock CE, Molyneux AJ, Rothwell PM, Oxford Vascular Study (2007). Change in incidence and aetiology of intracerebral haemorrhage in Oxfordshire, UK, between 1981 and 2006: a population-based study.. Lancet Neurol.

[45] Madsen TE, Khoury J, Alwell K, Moomaw CJ, Rademacher E, Flaherty ML (2017). Sex-specific stroke incidence over time in the Greater Cincinnati/Northern Kentucky Stroke Study.. Neurology.

[46] van Asch CJ, Luitse MJ, Rinkel GJ, van der Tweel I, Algra A, Klijn CJ (2010). Incidence, case fatality, and functional outcome of intracerebral haemorrhage over time, according to age, sex, and ethnic origin: a systematic review and meta-analysis.. Lancet Neurol.

[47] Béjot Y, Mehta Z, Giroud M, Rothwell PM (2013). Impact of completeness of ascertainment of minor stroke on stroke incidence: implications for ideal study methods.. Stroke.

[48] Ornello R, Degan D, Tiseo C, Di Carmine C, Perciballi L, Pistoia F (2018). Distribution and temporal trends from 1993 to 2015 of ischemic stroke subtypes: a systematic review and meta-analysis.. Stroke.

