# Supplementary material for: Trends in Stroke Incidence in High-Income Countries in the 21st Century: Population-Based Study and Systematic Review
Source: Stroke. 2020 Mar 25;51(5):1372–80. doi: 10.1161/STROKEAHA.119.028484 (PMC7185053; doi:10.1161/STROKEAHA.119.028484)
Supplement: Supplementary file 1 [file str-51-1372-s001.pdf]

Trends in stroke incidence in high-income countries in the 21st century: population-based study and systematic review

Linxin Li DPhil, **Catherine A Scott** BM BCh, **Peter M. Rothwell** FMedSci

On behalf of the Oxford Vascular Study

Centre for Prevention of Stroke and Dementia, Nuffield Department of Clinical Neuroscience, University of Oxford, Oxford, United Kingdom

SUPPLEMENTAL MATERIAL

- **Figure I&II, Table I**
- **Supplementary methods**
- **Supplemental references**

Figure I PRISMA flow diagram

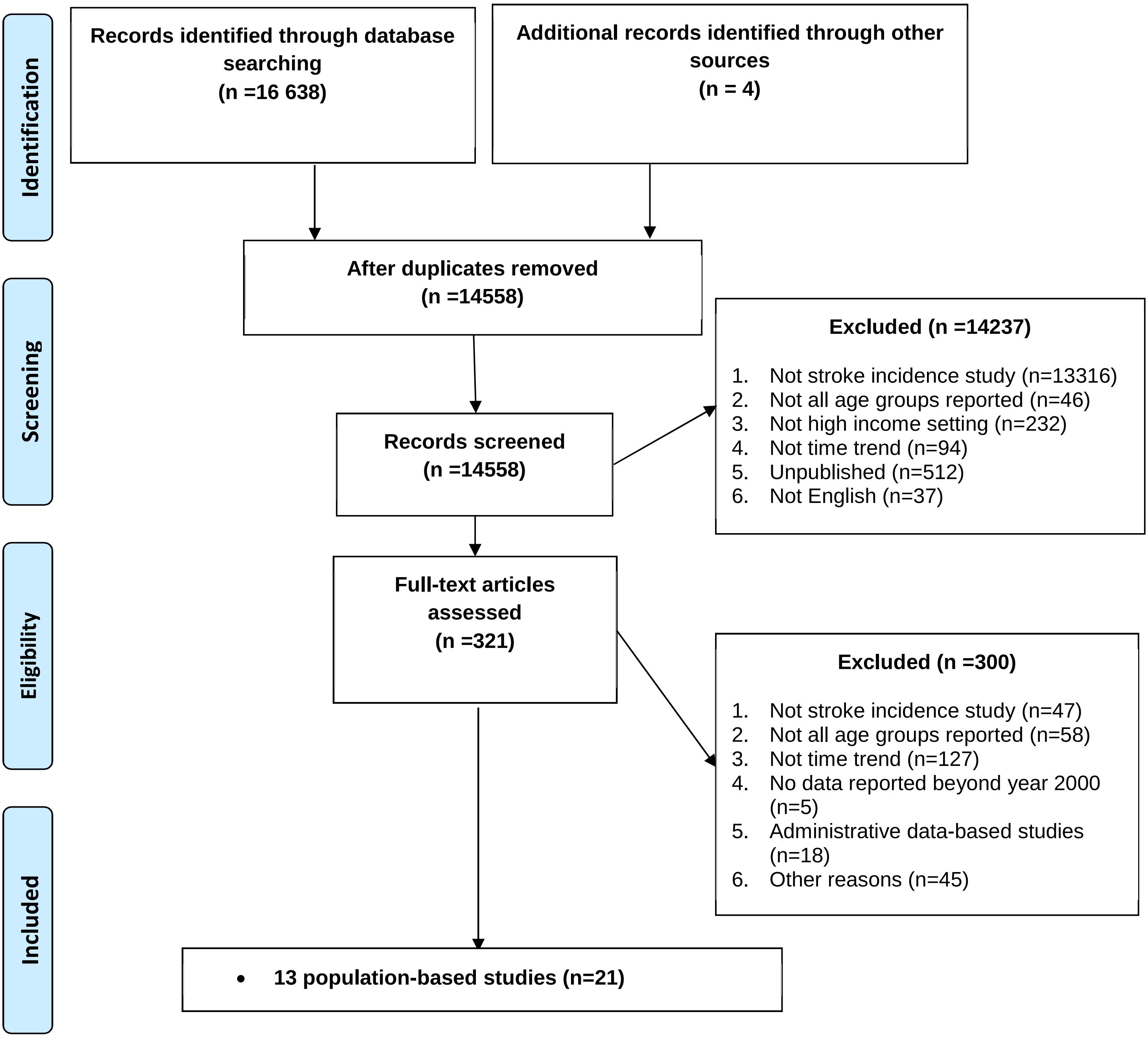

Table I Study characteristics and incidence (95%CI) of first-ever stroke (per 100,000 population) in additional population-based studies included in subgroup analyses stratified by study periods

Study (country)	Study duration	No. Stroke	Person-years	Age range	Crude incidence	Standardised incidence‡	Standardised population‡	Restandardised to European population†
**Valley d'Aosta, Italy[1,2]**	1989	254	114325	All	222 (195-249)	177 (138-215);240 (214-266)	European; Italian	177 (138-215)
	2004-2005	553	247496	All	223 (197-249)	126 (106-146);97 (80-114)	European; WHO	116 (106-126)
	2004-2008	1326	625515	All	212 (201-223)	189 (179-200);80 (73.2-87.2)	Italian; WHO	120 (113-127)
**Ludwigshafen, Germany[3,4]**	2006-2007	725	335812	All	216 (200-232)	146 (135-157)	European	146 (135-157)
	2006-2010	1779	838285	All	212 (199-225)	NR	NR	142 (135-149)
**Tartu, Estonia[5]**	1991-1993	829	331899	All	250 (233-267)	230 (214-246)	European	232 (216-248)
	2001-2003	451	202244	All	223 (203-245)	188 (171-207)	European	188 (170-206)
**Perth, Australia[6]**	1995-1996	213	136095	≥15y	157 (135-177)	92 (80-106)	WHO	144 (124-164)
	2000-2001	183	143417	≥15y	128 (109-146)	74 (63-86)	WHO	114 (97-131)

‡ reported in original publication; † Restandardised to European population based on raw numbers provided in original publication.

Figure II Meta-analysis (random effects) of standardised incidence rate ratio (temporal trend) in population-based studies of first-ever stroke stratified by study periods

A. 1990-2000†

Study	Study Duration	Time periods	IRR	95% CI
Oxfordshire, UK	20 years	2002-2005 vs. 1981-1986	0.80	0.70-0.91
Aosta Valley, Italy	15 years	2004-2005 vs. 1989	0.66	0.61-0.71
Auckland, NZ	11 years	2002-2003 vs. 1991-1992	0.90	0.83-0.97
Tartu, Estonia	10 years	2001-2003 vs. 1991-1993	0.81	0.72-0.91
Dijon, France	9 years	1994-2002 vs. 1985-1993	1.08	1.02-1.15
Lund, Sweden	7 years	2001-2002 vs. 1993-1995	0.91	0.82-1.02
Perth, Australia	5 years	2000-2001 vs. 1995-1996	0.79	0.65-0.97
Erlangen Germany	5 years	2000-2001 vs. 1995-1996	0.80	0.64-0.99
South London, UK	4 years	1999-2002 vs. 1995-1998	0.84	0.77-0.92
**Overall**	9 years		**0.84**	**0.74-0.94**

p=0.003
p_{het}<0.0001

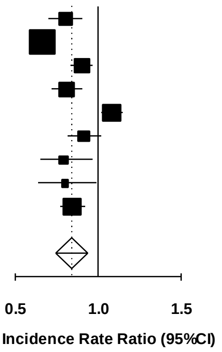

B. Post Year 2000‡

Study	Study Duration	Time periods	IRR	95% CI
Orebro, Sweden	18 years	2017 vs. 1999	0.50	0.42-0.59
Lund, Sweden	14 years	2015-2016 vs. 1993-1995	0.68	0.59-0.78
Martinique, France	13 years	2011-2012 vs. 1998-1999	0.68	0.60-0.77
Oxfordshire, UK	12 years	2014-2017 vs. 2002-2005	0.85	0.74-0.98
Porto, Portugal	11 years	2009-2011 vs. 1998-2000	0.76	0.66-0.87
Dijon, France	10 years	2003-2011 vs. 1994-2002	1.10	1.02-1.18
Auckland, NZ	9 years	2011-2012 vs. 2002-2003	0.84	0.78-0.90
South London, UK	8 years	2007-2010 vs. 1999-2002	0.76	0.69-0.83
Erlangen, Germany	8 years	2009-2010 vs. 2001-2002	0.98	0.86-1.12
Aosta Valley, Italy	3 years	2006-2008 vs. 2004-2005	0.93	0.83-1.05
Ludwigshafen, Germany	2 years	2006-2010 vs. 2006-2007	0.98	0.89-1.07
**Overall**	10 years		**0.82**	**0.72-0.93**

p=0.001
p_{het}<0.0001

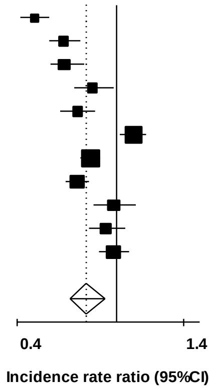

IRR=Incidence Rate Ratio. † Sensitivity analysis excluding Oxford, Perth, Erlangen and South London: IRR=0.86 (95%CI 0.71-1.04, p=0.13) for a mean study duration of 10 years. ‡ Sensitivity analysis excluding Aosta Valley and Ludwigshafen: IRR=0.80 (95%CI 0.70-0.92, p=0.001) for a mean study duration of 11 years.

Supplementary methods for the Oxford Vascular Study

Study population

The Oxford Vascular Study (OXVASC) is a prospective, population-based cohort study of all incident acute vascular events in all territories (transient ischaemic attack, stroke, acute coronary and peripheral vascular events).

The study population consisted of all 92,728 individuals, irrespective of age, registered with 100 general practitioners (GPs) in nine general practices in Oxfordshire, UK. In the UK, general practices provide primary health care for registered individuals and hold a lifelong record of all medical consultations (from the National Health Service [NHS] and private health care), and details of treatments, blood pressure, and investigations. In Oxfordshire, an estimated 97% of the true residential population is registered with a general practice, with most non-registered individuals being young adults. All participating practices held accurate age-sex patient registers, and allowed regular searches of their computerised diagnostic coding systems. The practices had all collaborated on a previous population-based study, for which they were originally selected to be representative of the urban and rural mix and the deprivation range of Oxfordshire as a whole.[7] Based on the index of multiple deprivation (IMD), the population was less deprived than the rest of England, but had a broad range of deprivation.

The OXVASC population is 94% white people, 3% Asian, 2% Chinese, and 1% Afro-Caribbean.[8] The proportion of whites is similar to that of the UK as a whole (88% white) and to many other western countries (Australia - 90%; France - 91%; Germany - 93.9%).

Case ascertainment

After a 3-month pilot study, the study started on April 1, 2002, and is ongoing. Ascertainment combined prospective daily searches for acute events (hot pursuit) and retrospective searches of hospital-care and primary-care administrative and diagnostic coding data (cold pursuit).

Hot pursuit was based on:

1) A daily (weekdays only), urgent open-access “TIA clinic” to which participating general practitioners (GPs) and the local accident and emergency department (A&E) send all individuals with suspected TIA or stroke whom they would not normally admit to hospital, with alternative on-call review provision at weekends. Patients too frail to attend are assessed at their residence by a study nurse or doctor.
2) Daily searches and case note review of admissions to the Emergency Assessment Unit, Medical Short Stay Unit, Coronary Care Unit and Cardiothoracic Critical Care Unit, Cardiology, Cardiothoracic, and Vascular Surgery wards, Acute Stroke Unit, Neurology ward and all other general wards when indicated.
3) Daily searches of the local A&E and eye hospital attendance registers.
4) Daily identification via the Bereavement Office of patients dead on arrival at hospital or who died soon after.
5) Daily searches of lists of all patients from the study population in whom a troponin-I level had been requested.
6) Daily assessment of all patients undergoing diagnostic coronary, carotid and peripheral angiography, angioplasty, stenting or vascular surgical procedures in any territory to identify both total burden of vascular invention and any potential missed prior acute events.

Cold pursuit procedures were:

1) Frequent visits to the study practices and monthly searches of practice diagnostic codes.
2) Monthly practice-specific list of all patients admitted to all acute and community NHS hospitals.
3) Monthly listings of all referrals for brain or carotid imaging studies performed in local hospitals.
4) Monthly reviews of all death certificates and coroners reports to review out-of-hospital deaths.
5) Practice-specific listings of all ICD-10 death codes from the local Department of Public Health.

Patients found on GP practice searches who have an event whilst temporarily out of Oxfordshire are included, but visitors who were not registered with one of the study practices are excluded. A study clinician assessed patients as soon as possible after the event in the hospital or at home. Informed consent was sought, if possible, or assent was obtained from a relative.

Data is collected using event-specific forms, for TIA and stroke, acute coronary syndrome or acute peripheral vascular events. Standardised clinical history and cardiovascular examination are recorded. Information recorded from the patient, their hospital records and their general practice records includes details of the clinical event, medication, past medical history, all investigations relevant to their admission (including blood results, electrocardiography, brain imaging and vascular imaging-duplex ultrasonography, CT-angiography, MR-angiography or DSA) and all interventions occurring subsequent to the event.

If a patient died before assessment, we obtained an eyewitness account of the clinical event and reviewed any relevant records. If death occurred outside the hospital or before investigation, the autopsy result was reviewed. Clinical details are sought from primary care physicians or other clinicians on all deaths of possible vascular aetiology.

All surviving patients are followed-up face-to-face at 1, 6, 12, 60 and 120 months after the initial event by a research nurse or physician and all recurrent vascular events were recorded together with the relevant clinical details and investigations. If face-to-face follow up is not possible, telephone follow-up is performed or enabled via the general practitioner. All recurrent vascular events that presented to medical attention would also be identified acutely by ongoing daily case ascertainment within OXVASC. If a recurrent vascular event was suspected at a follow-up visit or referred by the GPs to clinic or admitted, the patient was re-assessed and investigated by a study physician.

Definition of diagnosis

Although new definitions for stroke and TIA have been suggested recently,[8,9] in order to enable comparison with previous studies, the classic definitions of TIA and stroke are used throughout.[10] A stroke is defined as rapidly developing clinical symptoms and/or signs of focal, and at time global (applied to patients in deep coma and to those with subarachnoid haemorrhage), loss of brain function, with symptoms lasting more than 24 hours or leading to death, with no apparent cause other than that of vascular origin.[10] A TIA is an acute loss of focal brain or monocular function with symptoms lasting less than 24 hours and which is thought to be caused by inadequate cerebral or ocular blood supply as a result of arterial thrombosis, low flow or embolism associated with arterial, cardiac or haematological disease.[10] With the high rate (97%) of imaging or autopsy in OXVASC, strokes of unknown type were coded as ischaemic. Similarly, we also kept the same definitions throughout the study for acute coronary events and peripheral vascular events. Acute coronary events are defined using published criteria[11] based on available history, electrocardiography (ECG) findings, cardiac biomarkers (mainly troponin I), and autopsy or death certificate. Non-ST elevation (NSTEMI) and ST-elevation myocardial infarction (STEMI) are defined using standard criteria.[12] Sudden cardiac deaths are coded according to recent recommendations for epidemiological studies,[13] and required a definite history of preceding symptoms consistent with acute coronary ischaemia, or post-mortem evidence of either significant coronary atherosclerosis or acute thrombosis, or a documented myocardial infarction during the previous 28 days.[11] Sudden deaths were coded as probable cardiac deaths in the absence of the above characteristics if the person had a past history of ischaemic heart disease. Acute peripheral arterial events are defined as those affecting a limb or an organ other than the heart or the brain/eye. Aortic events included ruptured or acutely symptomatic aortic aneurysm or dissection.

Supplementary methods for the Oxfordshire Community Stroke Project

Study population

The Oxfordshire Community Stroke Project (OCSP) is a prospective population-based study of first-ever stroke and transient ischaemic attack (TIA).[14]

The study population comprised all patients registered with about 50 general practitioners (GPs) based in 10 general practices in Oxfordshire, UK, 8 of which overlaps with the study general practices of the Oxford Vascular Study. Registration of patients began on 01/11/1981 and continued until 31/10/1986. Partly due to problems in obtaining continuous funding and partly to allow interim analyses to be performed, the registration of cases of stroke was suspended between 01/11/1984 and 31/10/1985, during with time the follow-up of patients already in the study continued and the ascertainment of TIA cases was also continued.

Case ascertainment

Multiple sources were used for case ascertainment in OCSP. First, the collaborating GPs notified the study office as soon as possible of any patient with suspected acute cerebrovascular event. Second, a liaison GP in each practice regularly checked with colleagues to ensure that all relevant patients had been referred. Third, research nurses visited each practice at least once a week and a monthly newsletter was sent to all collaborators and GPs received a report on each of their patients referred to the study. Fourth, the admission and casualty registers of each hospital in the Oxford area were checked frequently without their GPs knowledge. The Oxford Record Linkage Study[15] enabled all patients with a given diagnosis who died in, or were discharged from, a hospital in the Oxford region to be identified. They also provided lists of patients with diagnoses coded to the International Classification of Diseases (9th Revision) rubrics 430 to 438 inclusive and rubric 342. Finally, copies of the death certificate of all residents of Oxfordshire were examined and copies of necropsy reports were also obtained.

Patients found on GP practice searches who have an event whilst temporarily out of Oxfordshire are included, but visitors who were not registered with one of the study practices are excluded.

All patients were assessed as soon as possible after the index event by a study neurologist, either in the hospital, in the outpatient clinic or at home. A standard neurological history and examination was performed. The GP and the hospital medical records of the patients were reviewed to confirm details of past medical history. If a patient died before the face-to-face interview, an eye witness account was obtained and information recorded in the GP and hospital notes was used. Computed tomographic (CT) scan or necropsy on every patient was attempted to establish the pathological subtype.

All cases were followed prospectively by research nurses at 1 month, 6 month, 12 month and then annually from the time of the stroke, using a standardised questionnaire to detect recurrent cerebrovascular and cardiovascular events. If a recurrent stroke was suspected, the study neurologist re-examined the patient. In addition, details of the use of health care resources and placement were recorded throughout the study. If the patient died, all available medical records were reviewed and a necropsy obtained whenever possible to ascertain the cause of death.

Definition of diagnosis

Clinical definitions of TIA and stroke are used throughout.[10] A stroke is defined as rapidly developing clinical symptoms and/or signs of focal, and at time global (applied to patients in deep coma and to those with subarachnoid haemorrhage), loss of brain function, with symptoms lasting more than 24 hours or leading to death, with no apparent cause other than that of vascular origin.[10] A TIA is an acute loss of focal brain or monocular function with symptoms lasting less than 24 hours and which is thought to be caused by inadequate cerebral or ocular blood supply as a result of arterial thrombosis, low flow or embolism associated with arterial, cardiac or haematological disease.[10]

Supplemental references (references 1-6 are cited in the main paper)

1. Corso G, Bottacchi E, Giardini G, De la Pierre F, Meloni T, Pesenti Campagnoni M, et al. Community-based study of stroke incidence in the valley of aosta, italy. Care-cerebrovascular aosta registry: Years 2004-2005. Neuroepidemiology. 2009;32:186-195
2. Corso G, Bottacchi E, Giardini G, Di Giovanni M, Meloni T, Pesenti Campagnoni M, et al. Epidemiology of stroke in northern italy: The cerebrovascular aosta registry, 2004-2008. Neurol Sci. 2013;34:1071-1081
3. Palm F, Dos Santos M, Urbanek C, Greulich M, Zimmer K, Safer A, et al. Stroke seasonality associations with subtype, etiology and laboratory results in the ludwigshafen stroke study (lusst). Eur J Epidemiol. 2013;28:373-381
4. Palm F, Urbanek C, Rose S, Buggle F, Bode B, Hennerici MG, et al. Stroke incidence and survival in ludwigshafen am rhein, germany: The ludwigshafen stroke study (lusst). Stroke. 2010;41:1865-1870
5. Vibo R, Korv J, Roose M. The third stroke registry in tartu, estonia: Decline of stroke incidence and 28-day case-fatality rate since 1991. Stroke. 2005;36:2544-2548
6. Islam MS, Anderson CS, Hankey GJ, Hardie K, Carter K, Broadhurst R, et al. Trends in incidence and outcome of stroke in perth, western australia during 1989 to 2001: The perth community stroke study. Stroke. 2008;39:776-782
7. Bamford J, Sandercock P, Dennis M, Burn J, Warlow C. A prospective study of acute cerebrovascular disease in the community: the Oxfordshire Community Stroke Project--1981-86. 2. Incidence, case fatality rates and overall outcome at one year of cerebral infarction, primary intracerebral and subarachnoid haemorrhage. J Neurol Neurosurg Psychiatry 1990;53:16-22.
8. Easton JD, Saver JL, Albers GW, Chaturvedi S, Feldmann E, Hatsukami TS, et al. Definition and evaluation of transient ischemic attack: a scientific statement for healthcare professionals from the American Heart Association/American Stroke Association Stroke Council; Council on Cardiovascular Surgery and Anesthesia; Council on Cardiovascular Radiology and Intervention; Council on Cardiovascular Nursing; and the Interdisciplinary Council on Peripheral Vascular Disease. The American Academy of Neurology affirms the value of this statement as an educational tool for neurologists. Stroke 2009;40:2276-2293.
9. Sacco RL, Kasner SE, Broderick JP, Caplan LR, Connors JJ, Culebras A, et al. An updated definition of stroke for the 21st century: a statement for healthcare professionals from the American Heart Association/American Stroke Association. Stroke 2013;44:2064-2089.
10. Hatano S. Experience from a multicentre stroke register: a preliminary report. Bulletin of the World Health Organization 1976;54:541-553.
11. Luepker RV, Apple FS, Christenson RH, Crow RS, Fortmann SP, Goff D, et al. Case definitions for acute coronary heart disease in epidemiology and clinical research studies: a statement from the AHA Council on Epidemiology and Prevention; AHA Statistics Committee; World Heart Federation Council on Epidemiology and Prevention; the European Society of Cardiology Working Group on Epidemiology and Prevention; Centers for Disease Control and Prevention; and the National Heart, Lung, and Blood Institute. Circulation 2003;108:2543-2549.
12. Alpert JS, Thygesen K, Antman E, Bassand JP. Myocardial infarction redefined--a consensus document of The Joint European Society of Cardiology/American College of Cardiology Committee for the redefinition of myocardial infarction. J Am Coll Cardiol 2000;36:959-969.
13. Coull AJ, Silver LE, Bull LM, Giles MF, Rothwell PM, Oxford Vascular S. Direct assessment of completeness of ascertainment in a stroke incidence study. Stroke 2004;35:2041-2045.
14. Bamford J, Sandercock P, Dennis M, Warlow C, Jones L, McPherson K, et al. A prospective study of acute cerebrovascular disease in the community: The oxfordshire community stroke project 1981-86. 1. Methodology, demography and incident cases of first-ever stroke. J Neurol Neurosurg Psychiatry. 1988;51:1373-1380
15. Acheson ED. In: Medical Record Linkage. London: OUP, 1967.
